# Review: 3-Aminopropyltriethoxysilane (APTES) Deposition Methods on Oxide Surfaces in Solution and Vapor Phases for Biosensing Applications

**DOI:** 10.3390/bios13010036

**Published:** 2022-12-27

**Authors:** Marzhan Sypabekova, Aidan Hagemann, Donggee Rho, Seunghyun Kim

**Affiliations:** 1 Department of Electrical & Computer Engineering, Baylor University, Waco, TX 76798, USA; 2 Center for Nano Bio Development, National NanoFab Center (NNFC), Daejeon 34141, Republic of Korea

**Keywords:** APTES, monolayer, functionalization, silanization, biosensor, oxide surface

## Abstract

Surface functionalization and bioreceptor immobilization are critical processes in developing a highly sensitive and selective biosensor. The silanization process with 3-aminopropyltriethoxysilane (APTES) on oxide surfaces is frequently used for surface functionalization because of beneficial characteristics such as its bifunctional nature and low cost. Optimizing the deposition process of the APTES layer to obtain a monolayer is crucial to having a stable surface and effectively immobilizing the bioreceptors, which leads to the improved repeatability and sensitivity of the biosensor. This review provides an overview of APTES deposition methods, categorized into the solution-phase and vapor-phase, and a comprehensive summary and guide for creating stable APTES monolayers on oxide surfaces for biosensing applications. A brief explanation of APTES is introduced, and the APTES deposition methods with their pre/post-treatments and characterization results are discussed. Lastly, APTES deposition methods on nanoparticles used for biosensors are briefly described.

## 1. Introduction

3-aminopropyltriethoxysilane (APTES) is an organosilane molecule that is frequently used in silane-based functionalization processes that attach biomolecules to surfaces. Organosilanes are silicon-based molecules that have a general formula of R′(CH_2_)nSi(OR)_3_, where R′ is an organofunctional group and R is a hydrolyzable alkoxy group [1,2]. Various organosilanes have been used for surface modification and functionalization, such as 3-aminopropyltriethoxysilane (APTES), 3-aminopropyltrimethoxysilane (APTMS), N-(2-aminoethyl)-3-aminopropyltriethoxysilane (AEAPTES), N-(2-aminoethyl)-3-aminopropyltrimethoxysilane (AEAPTMS), N-(6-aminohexyl) aminomethyltriethoxysilane (AHAMTES), 3-aminopropyldimethylethoxysilane (APDMES), 3-mercaptopropyltrimethoxysilane (MPTMS), glycidyloxypropyl-trimethoxysilane (GOPS), etc. Depending on the type of silane molecule used, the surface after the silane treatment can be aminated (e.g., APTES, APTMS, AEAPTES, AEAPTMS, and AHAMTES), thiolated (MPTMS) [3] or epoxy-group functionalized (GOPS) [4].

Among all organosilanes, APTES is the most used silane molecule to functionalize oxide surfaces [5,6,7,8,9,10,11]. Oxide surfaces are surfaces that have hydroxyl groups (-OH) bearing high surface energy that can rapidly interact and form a covalent bond with silane molecules [12,13,14,15]. Due to this strong covalent bond, bioreceptors can be chemically attached to the silane layer via charge-to-charge interactions or bifunctional crosslinkers without disrupting the silane structure [16,17]. The most extensively studied oxide surfaces contain Si-OH and Me-OH (Me: metal) groups. Because of their morphologic versatility, chemical stability, and physicochemical interfacial properties, oxide materials have been used extensively in biosensor development [18].

Biosensors are widely investigated and have been used in different areas such as environmental monitoring, healthcare (e.g., diagnostics, and point of care monitoring), security, food control, pharmaceuticals (e.g., drug discovery), and forensics. For environmental monitoring, the use of nanomaterials based on oxides for gas sensing has been widely exploited [19,20]. For example, metal oxide nanoparticles were used to absorb methane gas which reacted with oxygen ions and caused a decrease in resistance and, hence, detection [21,22,23]. For healthcare applications, biosensors are used to detect the presence and/or concentration of a biological analyte in complex biological media such as body fluids (e.g., blood, saliva, and urine), cell culture media, food, and environmental samples. Such information contributes to the formation of key medical decisions [4,24,25,26,27,28,29,30]. A typical biosensor consists of three main parts: an interface functionalized with the bioreceptor where a specific biological event takes place, a transducer that converts a biological event into a measurable signal, and a detector that amplifies and processes the signal to be displayed. Bioreceptors can be immobilized on the interface surface either physically (i.e., adsorption, encapsulation, electrostatic, van der Waals, and hydrophobic interactions) or chemically (i.e., covalent bonding, and crosslinking). There are different types of bioreceptors available, and they can be generally classified into five major categories: (1) antibody/antigen [31,32,33], (2) enzymes [34,35,36], (3) nucleic acids (DNA, RNA) [37,38], (4) cells and cellular structures [39], and (5) polymers [1,40,41,42]. However, most bioreceptors (except for polymers) cannot bind to the sensor surface directly; therefore, the surface first needs to be coated with a metal layer or covered with reactive molecules that provide binding sites for the bioreceptor. In this scenario, APTES molecules serve as an intermediate layer linking the oxide surface to bioreceptor molecules.

There are certain strategies that need to be followed to immobilize bioreceptors on oxide surfaces such as a surface pre-treatment, intermediate layer coating, and surface post-treatment processes. Prior to the surface pre-treatment steps, some native -OH groups may exist on the oxide surface, while the number of -OH groups can be increased by rehydroxylation [43]. Such hydroxylated surfaces have an increased number of attachment points for APTES molecules. For biosensor performance, the formation of an APTES monolayer is more desirable than the formation of a multilayer [9,10,44,45]. Thick layers of silane molecules have a very fragile structure [8,44] and get washed away either in the presence of a buffer or during various washing steps in an immunoassay process [5,10,45] leading to a nonuniform and inhomogeneous surface [31]. Additionally, for some biosensors (e.g., evanescent field-based sensors) whose response is affected by the depth of the detection layer, a thicker APTES multilayer could limit the sensor performance. The depth of the detection layer range of such sensors is from 50 to 200 nm [46,47,48] while an APTES multilayer thickness can reach up to 140 nm [14]. Therefore, the thickness of the layers from the functionalization steps, including the APTES and bioreceptor layers, could be beyond the detection layer range with which a sensor could function properly to detect the target analytes reliably.

The formation of a stable APTES monolayer is an important step for immobilizing bioreceptors well on an APTES-treated surface, otherwise the researchers developing biosensors might have a challenge knowing whether their measured signal change comes from the receptor/analyte binding or the unstable intermediate layer. This has a dramatic effect, therefore, on the overall biosensor performance including the limit-of-detection, stability, and reproducibility. However, the formation of a silane monolayer in most studies has been based mostly on conviction rather than experimental proof [9]. The ideal condition for obtaining monolayers of APTES on oxide surfaces is still unclear, and most studies have obtained results through trial and error by changing the reaction conditions [8]. Although the stability, durability, and repeatability of the immobilization, retention of antibody activity, and avoidance of nonspecific binding have been studied, it is uncertain if a universally applicable surface functionalization protocol for oxide silanization can be achieved [49]; therefore, researchers must determine the best silanization process for their specific applications and structures. To do so, it is important to understand the silanization processes that are reported to produce a monolayer of APTES with experimental proof. It is also important to understand the chemistry, conditions, and specific steps behind the APTES monolayer formation process because these conditions affect the thickness and roughness of the silane layer and the nature of the bonds created between the silane molecules and the oxide surface [9,45].

The purpose of this review paper is to provide a comprehensive guide for creating stable APTES monolayers based on the systematic analysis of research strategies developed for APTES monolayer fabrication, and to highlight the importance of this process to a broader research field. For that matter, in this review, we discuss the complex kinetics of APTES grafting and summarize the processes used for APTES monolayer formation confirmed with surface characterization parameters and detection results. We attempted to include the factors that affect the APTES monolayer quality, such as the deposition method, concentration of APTES, type of solvent used for the solution-phase deposition, deposition time, temperature, water content, pH, and the drying/curing conditions of the silane [11,44]. We have also included the stability of the APTES layer in a solution since this might have an immense impact on the overall biosensor performance. In addition, we briefly review the APTES deposition methods used for nanoparticles which have been actively investigated for biosensing applications.

## 2. APTES: Different Modes of Interaction with The Oxide Surface

**Figure 1:**
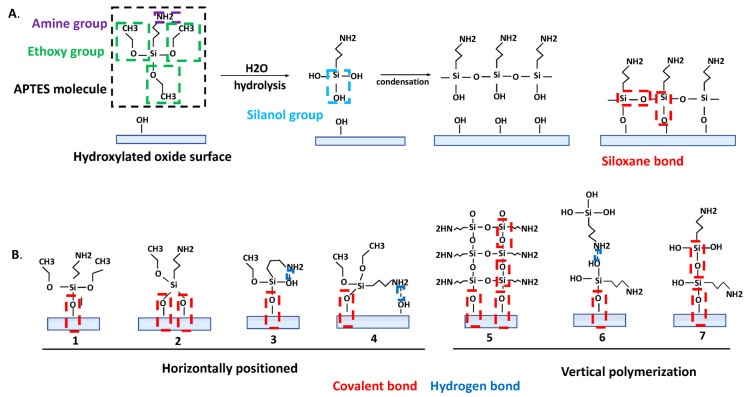
(**A**) Schematic representation of an APTES molecule, activation, and reaction with the hydroxylated oxide surface in an ideal situation. (**B**) Chemical interactions involved in APTES grafting on the hydroxylated oxide surface.

APTES has three functional reactive ethoxy groups and one amine group per one silane molecule (Figure 1). The polymerization of APTES molecules on an oxide surface is a very complex reaction involving three main steps: (1) hydrolysis (a hydroxyl group (-OH) substitutes the ethoxy groups in an acidic, alkaline, or neutral medium); (2) condensation (the formation of siloxane bonds (Si-O-Si)) (Figure 1); and (3) phase separation [8]. Due to the four reactive groups in APTES molecules and the complex kinetics in the three reaction steps, it is important to control and optimize the reaction conditions such as the environmental humidity, solvent type, amount of water, pH, reaction temperature and time, and silane concentration, to obtain a reproducibly smooth, stable, and high-density silane layer [8,10,45].

The quality of the silane surface, including its morphology, strongly depends on the initial hydrolysis step, making it very critical [50]. The water content determines the number of hydrolyzed groups during the hydrolysis step in the APTES molecule (hydrolysis of one, two, or three ethoxy groups) [51]. Ethoxy groups are very reactive toward water, forming silanol groups (Figure 1) [2]. In the absence of water or if the water content is too low, the silanol formation could be incomplete [10,44,52,53]. The optimal water concentration was suggested to be 0.15 mg of water in 100 mL of solvent [54], i.e., a water/silane ratio of 1.5 [8]. 

A condensation reaction can happen between two APTES molecules and/or between the oxide surface and an APTES molecule [55]. As the result of the condensation reaction between silanol groups on the APTES molecule and -OH groups on a pre-treated oxide surface or between neighboring hydrolyzed APTES molecules, the formation of siloxane bonds occurs (Figure 1A). Siloxane bonds can covalently attach the silane molecule to the surface in different ways (Figure 1B). In addition, the silanol groups of different silane molecules can interact with each other, thereby forming polymeric structures (Figure 1B(5–7)). This happens in the presence of excess water molecules and can cause the uncontrolled polymerization of aminosilanes [52,56,57,58,59], forming a polymer composed of polysiloxane, which can be seen as white specks on the surface [60,61]. 

In the phase separation step, the reaction medium loses its homogeneity to form more tangible polymeric structures [8]. Changing the temperature during APTES grafting can increase the overall reaction rate and changing the pH of the medium can dictate the different reaction kinetics. The amine group of the silane molecule is used to attach bioreceptors to the surface (Figure 1A); however, it can also form a weak hydrogen bond with the surface silanol group or with the silanol group of other silane molecules (Figure 1B). The formation of hydrogen bonds can decrease the number of silane molecules that are supposed to be covalently attached to the surface [62]. Additionally, the amine group can catalyze the formation and hydrolysis of siloxane bonds intramolecularly via the formation of stable five-membered cyclic intermediates (Figure 1B(4)) [44,61], and it can react with water, causing the release of nitrogen oxides, which results in the degradation of its reactivity [63]. 

In an ideal situation, the attachment of the bioreceptors on an oxide surface via silane molecules should start with the hydrolysis of the silane molecules followed by condensation without the formation of additional bonds or different reaction routes. To achieve this ideal case, one must tune the reaction conditions based on their laboratory facilities. According to the molecular structure, an APTES monolayer should have an average chain length of ∼5–10 Å [5,44,64,65], a 0.5–0.8 nm thickness [44,53,66,67,68], an average density of 2.1–4.2 molecules per nm^2^ [64,69] and a silanol group density of 5 per nm^2^ [43,50]. 

Due to the complex nature of APTES layer formation, great care should be taken to validate such surfaces for biosensing experiments. After the APTES grafting step, a post-deposition process (i.e., rinsing and baking) is usually performed to remove the non-covalently attached silane molecules without affecting the covalently-bonded silane molecules. The stability of the grafted APTES layer is another important aspect that needs attention in sensing experiments [70]. During the stability study, an APTES-treated surface is usually placed in a container with solution for an extended period (e.g., 1 to 24 h) to monitor the APTES desorption over time (i.e., the removal of physiosorbed molecules). Table 1 summarizes the methods used to inspect the APTES layer’s quality and stability.

## 3. Surface Preparation for APTES Deposition: Pre-Treatment Step

Before an APTES monolayer can be formed on an oxide surface, the surface must go through pre-treatment steps to remove organic contaminants and activate the sur-face with increased hydroxyl groups. Wet-chemical methods for the pre-treatment in-clude strongly oxidizing and acidic media such as “piranha solution” (i.e., 70% sulfuric acid (H2SO4): 30% hydrogen peroxide) [9,10,45,63,65,71] and hexavalent chromates in concentrated sulfuric acid [84] which can both be used to successfully clean and hydroxylate the surface of SiO_2_-based materials. Cleaning with 1:1 methanol/HCl removes the surface contaminants most effectively compared to various detergents and provides a smooth and clean surface for subsequent silane deposition [7,85]. The formation of silanol groups on SiO_2_-based materials using such pre-treatment steps are confirmed with FTIR results [14]. For metal oxide surfaces, an electrochemical passivation process called anodization is often used to clean the surface and increase the thickness of the natural oxide layer on it. When conducted in 1 M H2SO4 at various voltages, anodization can increase the number of -OH groups on the surface [86]. The activation of metal oxide surfaces can also be performed by placing them in a boiled 30% hydrogen peroxide solution for a certain period or by the aforementioned piranha cleaning process [63,72,87].

Exposure of the oxide surfaces to a dry-cleaning process, such as oxygen plasma and UV-ozone cleaning for a certain period (5–30 min), has also been used for surface cleaning and activation [70,80]. For example, X-ray photoelectron spectroscopy (XPS) revealed an increased number of -OH groups on oxygen plasma-treated surfaces relative to chemically-treated surfaces [88]. The oxygen plasma activation formed sufficient hydroxyl moiety resulting in a reduced surface roughness and the improved formation of a homogeneous layer [10,12,88].

Generally, after a sufficient cleaning and surface activation process, the oxide surface becomes hydrophilic with a water contact angle (WCA) ranging from ≤ 5 degrees [88] to 20–30 degrees [70,75] and a surface roughness of 0.45 nm [65]. An increase in hydrophobicity should be observed upon a silane layer deposition (with a WCA range of 40–65 degrees), and the hydrophobicity usually varies depending on the APTES deposition method, surface roughness, and compactness of the chemical chains on the surface [85,89]. The deposition of APTES on oxide surfaces can be completed in two different ways: via a solution where samples are dipped into the silane solution or via the vaporization of a silane solution onto samples. The distinguishing factor between these two methods is whether or not the APTES solution makes direct contact with the surface.

## 4. Solution-Phase APTES Deposition on Oxide Surfaces

**Figure 2:**
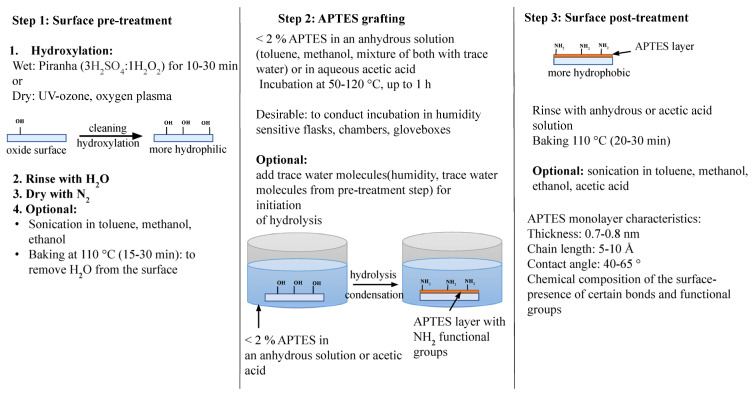
Solution-based APTES grafting on plain oxide surfaces. A general guideline for making APTES monolayers.

Among other silane deposition methods, solution-phase deposition is the most popular and easy to use. A solution-phase APTES deposition is performed by directly dipping the sample into an APTES solution and incubating it for a certain time. To make an APTES solution, APTES is dissolved in an anhydrous solvent (e.g., toluene, methanol, ethanol, or acetone) with or without a small amount of water [10,90]. Based on a systematic analysis of experimental procedures that mainly focused on creating an APTES monolayer, dissolving APTES at a certain ratio and for a certain incubation time in toluene, methanol and acetic acid produced a near-monolayer surface as compared to dissolving the APTES in ethanol. Nevertheless, it was also shown that it is possible to remove the physiosorbed APTES molecules from a multilayered surface such as those dissolved in ethanol by rinsing with acetic acid, thereby creating a monolayered surface. After incubation, as a post-treatment step, the sample was rinsed with an anhydrous solution or acetic acid to hydrolyze the ethoxy group and remove the unbound APTES molecules from the sensor surface. The surface was then baked at an elevated temperature (≥ 110 °C) for at least 30 min to cross-link the APTES on the surface further and remove the residuals. Figure 2 illustrates the steps used in the APTES grafting process based on studies which produced APTES monolayers and confirmed their stability with hydrolytic stability tests.

### 4.1. Anhydrous Solvent-Based APTES Deposition with Toluene

To avoid uncontrolled polymerization in the presence of water, the solution-phase deposition of APTES is generally undertaken in an anhydrous solution. Out of all the anhydrous solvents used for APTES deposition, toluene has been widely used [5,10,44,45,53,63,64,65,71,86].

Meroni et al. showed the monolayer formation of APTES with 2 M APTES in a 150 µl toluene solution on a TiO_2_ surface [64]. With 2 h of incubation under a dry N_2_ atmosphere in an oven at 80 °C, the authors reported the successful formation of a monolayer of APTES based on AFM topography with an RMS roughness of 0.75 nm. XPS results showed a thickness of ∼5.3–6.5 Å, comparable to the monolayer thickness. According to the XPS analysis, the APTES deposition on TiO_2_ resulted in 60% of free amine groups, and the other 40% were suggested to be involved in hydrogen bonding or protonated. This was also supported by FTIR results where peaks were observed for the free amine and terminal amine groups cross-linked with silanol groups. Near-edge X-ray absorption fine structure (NEXAFS) results supported the formation of a low-density monolayer with APTES molecules for hydrocarbon groups (i.e., one or two Si-O-Ti bonds involving the Si headgroup) tilted toward the surface and with no clear preferential orientation for N groups (i.e., conformationally freer than the whole hydrocarbon chain).

Another research study showed that 100 mM of APTES in a 50 mL toluene solution formed a monolayer of APTES on TiO_2_ [86]. The metal oxide-coated substrate was placed in a sealed flask and immersed in the solution from 0.5 to 24 h. The reaction was carried out in a temperature ranging from 25 to 90 °C. XPS data revealed that the deposition had reached its saturation point after 1–2 h with 100 mM of APTES. After 10 h of incubation, the TiO_2_ surface was completely covered with APTES multilayers, possibly due to vertical polymerization or agglomeration. The authors concluded that incubations for more than 8 h between 0.01–10 mM of APTES and incubations for 8 h at 10 mM of APTES created a stable monolayer without any tendency to form 3D networks.

Zhu et al. grafted APTES on silicon wafers by placing them in a sealed flask with 25 mL of toluene and 0.5 mL of APTES (1.98% APTES) at either 70 °C or 90 °C for 24 h [44]. The flask was constantly purged with N_2_ gas to control the humidity. Despite this, the authors stated that ambient humidity (e.g., 20% in winter and 60% in summer) had an immense impact on the APTES layer thickness. More humidity produced thicker and more varied APTES layers based on ellipsometry measurements (23 Å) and WCA measurements (32 degrees). In addition to the humidity, the prolonged incubation time (24 h) could have also played a role in forming thicker APTES. From a hydrolytic stability test, the authors found that the thickness decreased from 23 Å to 8 Å during 24 h of incubation in water, confirming the APTES’ instability. A similar study with the same reaction conditions (0.5 mL of APTES dissolved in 25 mL of toluene under N_2_ gas at 70 °C) resulted in a monolayer formation at 3 h of incubation with an ellipsometry-confirmed thickness of 10 Å and WCA 38–43 degrees; however, the APTES layer formed this way was not hydrolytically stable [5].

Howarter et al. showed that prolonged incubation times of 24–74 h resulted in thicker APTES layer formation, confirmed by ellipsometry measurements ranging between a 5–16.3 nm thickness [71]. The deposition of APTES, in this case, was conducted on silicon wafers in a closed container (i.e., a toluene solution, and an APTES concentration range of 1–33%) backfilled with N_2_ gas. Based on the AFM surface roughness (0.53 nm), ellipsometry (1.5 nm), WCA (60–68 degrees), and XPS measurements (C:N ratio of 5.5), the group concluded that 1% APTES dissolved in toluene and incubated for 1 h created a thinner and consistent APTES layer.

Studies conducted by Pasternack et al. confirmed that lower APTES concentrations (0.1%) dissolved in toluene could create densely packed propyl chains on SiO_2_ when the reaction was carried out at 70 °C for less than 1 h. This result was supported by ellipsometry thickness results of 1.8 nm, an AFM surface roughness of 0.3 nm, and additional XPS data on the presence of APTES-associated chemical bonds [53]. The authors concluded that this process produces 2–2.5 layers of APTES and reported a 50% degradation after 6 h of incubation in water.

According to Gunda et al., a 1 h incubation of 2% APTES dissolved in toluene created a layer with a thickness of 2.4 ± 1.4 nm (based on ellipsometry measurements) on the surface of silicon wafers when the reaction was carried out at 100–120 °C. The authors reported that the thickness was close to the reported thickness of a silane monolayer and that it had a WCA of 63 degrees and an AFM surface roughness of 0.69 nm [10].

Interestingly, Yadav et al. reported that the incubation of silicon wafers in 1% APTES in toluene resulted in two- and three-dimensional polymer networks at room temperature and 70 °C. The authors suggested that ambient humidity might cause such polymerization [65].

The desorption of APTES molecules from heat-treated APTES was found to be less and slower compared to an APTES incubation at room temperature At higher temperatures, the total amount of deposited silanes was less, but their structure was more uniform and hydrolytically stable (both in water and a buffer) [45,71].

### 4.2. Anhydrous Solvent-Based APTES Deposition with Ethanol

In addition to toluene, ethanol has also been used as an anhydrous solvent for APTES deposition.

Miranda et al. used 5% APTES in an anhydrous ethanol solution to treat a Si substrate for 20 min at room temperature [7]. XPS studies revealed that, compared to a deposition in toluene, the presence of N and C was three and two times greater, respectively, indicating more than one monolayer of APTES. Based on the calculated C:N ratio, increasing the APTES concentration and reaction time (from 20 to 60 min) did not significantly influence the multilayer formation. The authors attributed this finding to the polar protic nature of ethanol that can solvolyze the Si-O-Si bonds faster than the condensation reaction. After deposition, the non-covalently attached APTES molecules were removed using 6% acetic acid, effectively removing the multilayer and leaving the surface with an APTES monolayer, as proved by AFM and XPS surface analysis.

Han et al. confirmed the formation of an APTES multilayer on a SiO_2_ surface when samples were incubated in 5% APTES dissolved in an anhydrous ethanol for 20 min, 1 h, 3 h, and overnight at 50 °C [14]. The group showed that APTES tended to accumulate on the surface, resulting in a non-uniform layer with increasing thicknesses: 10 nm (20 min), 32 nm (1 h), 75 nm (3 h), and 140 nm (20 h). After 20 h of incubation, an increase of nitrogen up to 11.2% and of carbon up to 52.2% was observed from the XPS results, indicating a thick APTES multilayer formation. Anhydrous acetic acid was used for the rinse step after the deposition and 1 mM of acetic acid helped to activate the silane (i.e., forming a network on the surface) and to rinse off the weakly bound APTES from the surface, resulting in a thickness decrease from 140 nm to < 4 nm and decreases of nitrogen down to 5.1% and carbon down to 36.6% from the XPS measurements. The authors concluded the importance of acetic acid for forming the thinnest APTES layer.

One study determined the optimal concentration of APTES in a solution based on protein immobilization (mouse IgG) on the surface [9]. The IgG was detected through a reaction with a fluorescently labeled anti-mouse IgG antibody. Maximum plateau fluorescence signal values were obtained at ≥ 5% APTES dissolved in anhydrous ethanol. The reaction was carried out at room temperature, and 60 min of incubation and 20 min of a final cure at 120 °C were enough to reach the plateau. The authors also conducted an APTES stability test by measuring the fluorescence signal from the IgG immobilized surfaces over 2 months. They found that the fluorescence intensity remained the same for almost 3 weeks, indicating that the APTES layer was stable

### 4.3. Solution-Based APTES Deposition with Water Molecule Traces

As previously mentioned, the amount of water in a solution and native -OH groups on an oxide substrate is critical in controlling the level of polymerization of silane molecules. Some trace water can be added to the reaction solution, or it could be present in a solution, such as in 95.6% ethanol with 4.4% water.

Dissolving APTES in methanol with some water traces resulted in forming an APTES layer with 8.1 Å thickness based on spectroscopic ellipsometry measurements, which is close to the thickness of a monolayer [65]. A 1:500 ratio of stock solution (i.e., 50% methanol, 47.5% APTES, and 2.5% nanopure water) in methanol was used to deposit a monolayer of APTES. The thickness of the surface was retained by about 50–70% after the hydrolytic stability test of a 60 min incubation in water. The desorption of APTES molecules from the surface was negligible in buffer solutions such as PBS compared to water. The monolayer formation was further supported by AFM surface roughness tests at 0.2 nm and WCA at 45–60 degrees.

Arnfinnsdottir et al. optimized the silanization of SiO_2_ on a functioning sensor through the immobilization of proteins [49]. For the APTES deposition, the surface was incubated in 1–4% of APTES dissolved either in aqueous 1 mM of acetic acid or in 95% ethanol. AFM measurements produced a roughness of 0.1 nm for the APTES layer formed in the acetic acid, which was constant with higher APTES concentrations and various incubation times from 10 to 60 min. Based on the nanodomains formed after silanization, the authors concluded that the acetic acid solvent did not promote extensive APTES self-polymerization. A uniform layer could be formed with 1–2% of APTES with an incubation of 10–20 min. Additionally, the surface roughness (0.1–0.6 nm) and nanodomain number and sizes increased when the silanization was performed in 96% ethanol, indicating increased APTES self-polymerization. The data on protein detection showed that using acetic acid as the solvent in the silanization step generally yielded a higher protein binding capacity for C-reactive protein (CRP) onto anti-CRP than using ethanol.

## 5. Vapor-Phase APTES Deposition on Oxide Surfaces

**Figure 3:**
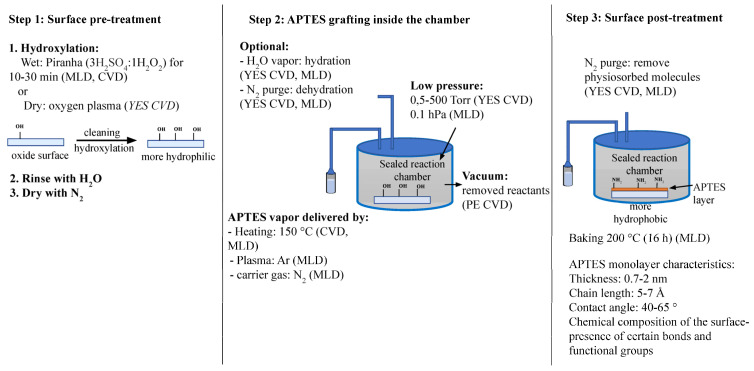
Vapor-phase APTES grafting on plain oxide surfaces. A general guideline for producing APTES monolayers.

The alternative to solution-phase APTES deposition is depositing APTES in the vapor-phase. Solution-phase deposition methods are generally preferred and have been more heavily researched because of their simplicity, but vapor-phase deposition methods remove the need for high-purity anhydrous solvents and offer more control over the deposition environment [67]. As previously explained, an excess amount of water is not desirable for APTES deposition; however, having a trace amount of water is important for hydrolysis of the APTES ethoxy groups and promoting siloxane bond formation. It has been reported that precise water content control when using anhydrous solvents is difficult because of variations in ambient humidity [44]. This negative effect is not a factor with vapor-based deposition, where solvents are not used. A vacuum or nitrogen purge can be used to further remove any unwanted moisture in the air or on the deposition surface [71]. Figure 3 illustrates the typical vapor-phase APTES deposition process. Step one remains mostly the same compared to the solution-phase methods, but in Steps two and three, the advantages of the vapor-phase are clear: no anhydrous solvents are required, sealed chambers with an optional vacuum are used to control the environment, and optional N_2_ purges remove physisorbed molecules. Lastly, note that there are no manual processes such as soaking, washing, and drying that can cause a variation in the film quality [66]. Vapor-phase deposition methods can be placed into two different categories: chemical vapor deposition (CVD) and molecular layer deposition (MLD), and both CVD and MLD have been used to produce high-quality, repeatable APTES monolayers onto silicon oxide surfaces.

### 5.1. Chemical Vapor Deposition (CVD)

Yield Engineering Systems (YES) CVD equipment can precisely control deposition parameters such as the reactant volume, reaction temperature, and deposition time, making them ideal for APTES deposition. Although they are bulky and expensive, the deposition process is relatively simple. The oxide surface can be hydroxylated with plasma using the YES system or a piranha solution beforehand. The APTES is stored in temperature-controlled flasks where it is evaporated and introduced to the deposition chamber, which is kept at 150 °C and a low pressure (0.5–500 Torr). Nitrogen purges have been used before the deposition to dehydrate the surface or after deposition to remove physisorbed molecules. Rather than dehydrate the surface, water vapor can also be introduced to hydrate it if so desired. The deposition time using a YES system is short compared to other methods. In the literature, YES CVD systems have been used to deposit monolayers of APTES using processes with different steps [65,67,91].

Zhang et al. utilized a dehydration purge to remove water from plasma-activated oxide surfaces. The deposition chamber of a YES CVD system was evacuated to 5 Torr, then filled with N_2_ to 500 Torr. Finally, they evacuated to 1 Torr [67]. Evaporated APTES was then introduced for 5 min, raising the pressure to 2–3 Torr. Finally, to remove residual silanes, the chamber was purged with three cycles of N_2_. Smooth monolayers of APTES were verified using AFM and ellipsometry, with a thickness of 0.66 nm and a roughness of 0.152 nm. Additionally, the APTES was desirably hydrophobic with a WCA of 44 degrees. Notably, neither the WCA nor the surface coverage was affected by the concentration of APTES used, which is an advantage over solution-based methods. Using XPS, the stability of the APTES layer was verified by measuring the presence of nitrogen before and after prolonged exposure to a pH 10 buffer. After two hours, the APTES had lost 20% of its nitrogen content, and after four hours, 30–35% had been lost, indicating that the deposited monolayer was stable for an extended period.

Another piece of research utilizing YES CVD used plasma-activated oxide surfaces, but instead of dehydrating them, they were hydrated with 500 L of water [65]. This preceded the ten minute deposition of 500 L of APTES at 500 Torr, after which several nitrogen purges were used to remove excess silane. With this process, smooth, hydrophobic APTES monolayers were produced with a thickness of 0.42 nm, a roughness of 0.22 nm, and a WCA of 40 degrees. When exposed to water for one hour to test the stability, 50–70% of the APTES layer remained intact.

Saini et al. used YES CVD to deposit monolayers of APTES onto oxide surfaces that were cleaned and hydroxylated with piranha solution without using hydration, dehydration, or N_2_ purges step [91]. An amount of 2 mL of APTES was introduced to the deposition chamber for 30 min at a base pressure of 0.5 Torr. The APTES monolayers were smooth and hydrophobic with a thickness of 0.65 nm, a roughness of 0.239 nm, and a WCA of 44 degrees.

The CVD of APTES monolayers can also be completed without using bulky machines or vacuums. In this case, samples are simply placed in a sealed container, and heat is used to vaporize liquid APTES inside. For a monolayer deposition on an oxide surface, Zhu et al. introduced nitrogen to a Schlenk flask in addition to 0.5 mL of APTES [44]. Deposition times of 24 and 48 h were tested, with two 24 h samples being completed at 70 and 90 °C and one 48 h sample completed at 90 °C. After the deposition, the samples were rinsed twice with toluene, ethanol, and water and dried at 110 °C for 15 min. For all three samples, a hydrophobic monolayer with a thickness between 0.5 and 0.6 nm was produced. At a 24 h deposition time, when the temperature was increased to 90 °C, the thickness decreased from 0.6 to 0.5 nm. At 90 °C, when the deposition time was doubled from 24 to 48 h, the thickness remained at 0.5 nm, and the WCA increased from 50 to 51 degrees. The thickness of all three APTES layers was reduced to 0.3 nm after 24 h in water. This thickness is indicative of a APTES sub-monolayer. This study also performed a solution-based APTES method with toluene, but this did not produce a monolayer. It was reported that the vapor-based method was less sensitive to variations in the reaction environment.

### 5.2. Molecular Layer Deposition (MLD)

Molecular layer deposition (MLD) is a relatively new method for depositing thin films of organic materials such as APTES. Generally, the distinguishing feature of MLD is the use of two precursors and the sequential, non-overlapping introduction of them to the deposition chamber. This pulsing of the two reactants forms self-limiting reactions on the surface. These self-limiting reactions are what make MLD beneficial for APTES deposition because they allow the process to be precisely controlled. Like YES CVD, there is some variation in the details of MLD processes, for example, they can be undertaken with or without high temperatures, a substrate dehydration, and nitrogen purges.

Liang et al. used an NCD Tech D100 MLD system to pulse H_2_O vapor and APTES simultaneously for 5 s, producing APTES monolayers on oxide surfaces hydroxylated via piranha solution [66]. The reaction temperature was 110 °C, nitrogen was used as the carrier gas (50 sccm), and after deposition, the chamber was purged with nitrogen for 60 s. For 5, 10, and 20 pulses, the thickness and WCA results were very similar: 1–1.2 nm and 55–61 degrees. After twelve hours in water, the APTES layer of all samples became a monolayer with thicknesses between 0.7 and 0.8 nm, and the WCA decreased to between 34 and 47 degrees. Roughness measurements also indicated a smooth surface with a roughness between 0.172 and 0.186 nm. The MLD process was compared to a thirty minute ethanol solution-based process. Although it also created stable monolayers after hydrolysis, the MLD process was ultimately preferred because the processing time was much shorter. The produced APTES coverage was better, as verified by a 15.8% higher sensitivity when using both processes to fabricate pH sensing devices.

Yuan et al. used only APTES as a precursor in their MLD process [68]. They simply introduced 0.3 mL of APTES via an argon carrier gas (27 sccm) to a deposition chamber containing the oxide surfaces, which were hydroxylated with oxygen plasma. A vacuum was used to maintain a process pressure of 0.375 Torr during the deposition, which lasted ten minutes. After deposition, the argon and APTES were removed, and the pressure was reduced to 75 × 10^−7^ Torr. This post-deposition step was found to be vital for forming stable APTES monolayers, as it allows for the removal of physisorbed molecules. When this step was 24 h long, a hydrophobic monolayer was produced with a thickness of 0.71 nm and a WCA of 63.9 degrees.

Table 2 summarizes all the referenced solution-phase and vapor-phase processes and their monolayer characterization results.

## 6. APTES Deposition Process on Oxide Nanoparticle Surfaces

The chemical reaction between the APTES and oxide nanoparticles’ (NP) surface is similar to the reaction with plain oxide surfaces. Coating the NPs with APTES permits various improvements in the NPs functionalities such as an antibacterial effect, photostability, and the attachment of other functional groups [15,92]. Most NPs coated with APTES are used in applications such as for chemical sensors, cosmetic products (e.g., UV scattering effect in sun cream), drug delivery, magnetic resonance imaging, and bioimaging [15]. The formation of APTES on NP surfaces, for example, has been used to change the agglomeration or physical properties of the particles.

In one study, Zinc Oxide (ZnO) NPs were coated with APTES dissolved in water and toluene. The pH of the water was adjusted to an acidic or basic environment [92]. The condensation reaction rate of the silanol into siloxane (Si-O-Si) appeared to be very dependent on the pH level. At an acidic pH, the hydrolysis process of silane is usually activated, whereas, with a basic pH, both hydrolysis and condensation are activated. Acidic and basic environment-based APTES deposition in that study was carried out by dispersing the NPs in 50 mL of water (with an adjusted pH), followed by adding 1 mL of APTES and stirring for 1 h. For a toluene-based APTES deposition, the NPs were dispersed in 200 mL of toluene and stirred for 1 h under an argon flow. In all three cases, the APTES played a role of an NP’s growth inhibitor, as confirmed by scanning electron microscope (SEM) images. The amount of Si measured by an inductively coupled plasma (ICP) atomic emission spectroscopy in the case of acidic and basic environments were 0.5 and 4.8%, respectively. More impurities were observed on APTES-deposited NPs in a basic environment. The amount of Si in the case of toluene was 0.3% due to the low water quantity present during the experiment (i.e., the majority of water was chemisorbed and physisorbed on the surface of the NPs).

The formation of more APTES monolayers on nanoparticles was proposed by Liu et al., where good quality silane layers were formed when the reaction was carried out at an elevated temperature using methanol/toluene mixed solvents [80]. The monolayer formation was based on calculating the silane grafting densities using thermal gravimetric analysis. The study revealed that the equilibrium adsorption capacity was 301.2 mg/g for the highest grafting density during incubations at 70 °C using 2% APTES. Moreover, the experimental grafting density was very close to the theoretically predicted APTES monolayer density. Additionally, APTES grafting on Fe_2_O_3_ magnetic NPs can prevent oxidative damage and play a role in the hydrophilicity or hydrophobicity of the NP surface, which is important for further drug conjugation through terminal amine groups.

Karade et al. conducted a ninhydrin colorimetric assay to monitor the monolayer formation through the quantification of the amine groups on the NPs [83]. The process was based on a nucleophilic displacement reaction among the primary amines and ninhydrin, where 1 g of Fe_2_O_3_ NPs were dispersed in 100 mL of a methanol/toluene mixture at a 1:1 ratio. The mixture was sonicated for 30 min, followed by heating at 95 °C until half of the solution was evaporated. This procedure was repeated three times and 2% APTES in 100 ml of methanol was used to cover the NPs surfaces with silane molecules. The total calculated fractional monolayer coverage based on the ninhydrin assay on 1 NP was 96.6%. The FTIR and TEM-EDX spectra of the APTES-modified NPs showed the presence of a stretching vibration of Fe-O-Si and the presence of Si, confirming the APTES coverage. XPS results revealed the presence of Fe, O, C, and N, consistent with the development of interlinking silane molecules on a NP’s surface.

A summary of the APTES deposition processes on oxide NPs is presented in Table 3.

## 7. Conclusions

The chemical structure of an APTES molecule (i.e., with four reactive groups) makes any interaction with an interface surface multidirectional and interchangeable (with intra- and inter-layer modes of coupling), incorporating both covalent and noncovalent (hydrogen bonds) binding. Due to the continuous bond–breaking–bond–reforming horizontal and vertical rearrangement of the siloxane groups, the formation of polymerized multilayers can be observed, which greatly affects the biosensor performance. In most cases, the possible formation of such multilayers and their instability in a reaction solution (e.g., a buffer, or biological media) is underestimated in the various biosensor development processes. Moreover, the same deposition methods carried out in different laboratories do not guarantee a repeatability of the APTES monolayer formation. This is because varied conditions, such as the environmental humidity, affect the APTES’ monolayer formation; therefore, the careful surface characterization and validation for a specified laboratory setup are important to determine the optimal APTES deposition process.

Solution-phase deposition on oxide surfaces and nanoparticles can be completed by dissolving APTES both in anhydrous solutions (e.g., toluene, ethanol, and methanol) and solutions with trace water molecules (e.g., ethanol, methanol, and acetic acid). The presence of some adsorbed water on the surface or in the APTES solvent was found to be important for initiating a hydrolysis reaction during the APTES formation process. Incubations at an elevated temperature (e.g., 50–120 °C) promote the formation of stable covalent siloxane bonds while disrupting hydrogen bonds. The solution-phase deposition methods create a multilayer on an oxide surface with a different thickness. More concentrated APTES solutions (>2%) result in a thick multilayer formation, whereas less concentrated solutions result in thinner APTES layers; therefore, a rinse step is required to remove most of the physisorbed silane molecules to obtain an APTES monolayer. The major limitation and challenge of solution-phase APTES deposition is that one must control the environmental humidity during the reaction process, as humidity has been shown to greatly affect the thickness of the layer in addition to maintaining the anhydrous condition for solutions. To create the most stable monolayer using the solution-phase deposition, it is suggested to initially deposit a thin APTES layer (i.e., with a thickness equivalent to 2–2.5 APTES layers) followed by the removal of physisorbed, weakly-bound silane molecules from the surface through rinsing. The hydrolytic stability test suggests conducting measurements within approximately 1–6 h for efficient detection.

Although it has been used less frequently, the vapor-phase deposition of APTES offers some advantages over a solution-phase approach. Because the amount of water in the deposition environment should be tightly controlled, using a vacuum is desirable as it can negate environmental factors such as the humidity and remove the need for perfectly anhydrous solutions. Not only can water be removed entirely via the vacuum, but special equipment can also be used to introduce a precise volume of water vapor to the deposition chamber, and controlled nitrogen purges can be used to remove excess silane. These benefits are taken better advantage of when using YES CVD and MLD, which generally include more expensive and sophisticated equipment. Both methods have been used to create reproducibly smooth and stable monolayers at shorter deposition times. YES CVD does not require lengthy post-treatment steps and it only takes 5–30 min to produce high-quality monolayers, while MLD processes have required hours-long post-treatment steps to remove non-covalently bonded APTES. Additionally, YES CVD offers some flexibility in the process steps since monolayers can be produced with or without hydration, de-hydration, or nitrogen purges. CVD without specialized equipment can be used to create high-quality monolayers, although the reaction time can be in the range of 24–48 h, and it requires a rinse process to remove any loosely bound multilayers. With that, the water content is not quite as easily controlled, but with nitrogen purges and a properly sealed container, the effects of humidity variation can be curtailed, and there is still the added benefit of avoiding anhydrous solutions which may not be perfectly pure. The main limitation and challenge of using a vapor-phase deposition of APTES is the unavailability of sophisticated lab equipment used to control the deposition process.

When comparing the two methods for producing APTES monolayers on oxide surfaces, namely, the solution-phase and vapor-phase deposition methods, vapor-phase deposition is better for reproducing results in different lab environments; however, for a lab without such specialized equipment, solution-phase methods or CVD processes without specialized equipment can still be used to achieve an APTES monolayer on an oxide surface for biosensing applications. To confirm the formation of an APTES monolayer, characteristics such as an APTES layer thickness in the range of 0.5–0.8 nm, a WCA range of 40–68 degrees, a surface roughness at 0.2–0.75 nm, and/or a quantitative atomic composition of the modified surface using XPS or FTIR can be used. For reliable and repeatable results, stability tests in a solution after the deposition are highly recommended to establish the period at which an APTES modified surface remains stable.

## Figures and Tables

**Table 1:** List of methods used for inspecting and confirming APTES monolayer and/or multilayer formation on oxide surfaces.

Method Name	Brief Description	Ref.
Ellipsometer	Calculates the thickness of each layer formed on the surface after each modification	[10,44,65,70,71,72]
Contact angle measurement	Quantitatively measures the wetting of a modified surface	[10,14,44,65,70]
Atomic force microscopy	Scans and acquires images of the modified surface, estimating the surface roughness	[10,14,45,65,71,73]
X-ray photoelectron spectroscopy (XPS)	Determines quantitative atomic composition and chemistry of the surface; quantitative analysis of the degradation process	[6,14,64,70,73,74]
Fluorescence microscopy	Visualizes the reporter molecules: Alexa Fluor, FITC that are specifically bound to the amine-modified surface	[10,50,70,74,75,76]
IR spectroscopy	Measures absorption, emission, and reflection of the modified surface and determines the functional groups in molecules	[77,78,79]
Near-edge X-ray absorption fine structure (NEXAFS)	Measures the absorption of an X-ray photon to analyze the matter density of a layer	[64]
Fourier transform infrared spectroscopy (FTIR)	Identifies the chemical composition of the modified surface	[6,10,14,74,80]
Zeta potential	Measures surface charges	[81]
Electrochemical	Measures the electronic transport at the electrode solution interface	[65,70,82]
Hydrolytic Stability test	Identifies the stability of the APTES in the presence of water/buffer	[10,44,65,71,72]
Transmission Electron microscope energy-dispersive X-ray spectroscopy (TEM-EDX)	Identifies the morphology of the particles and performs chemical characterization of the surface.	[80,83]

**Table 2:** APTES grafting methods and characterization results on oxide surfaces.

Oxide Surface	Solution vs Vapor Phase Deposition	Surface Pre-Treatment	APTES Deposition	Post-Treatment	APTES Monolayer Characterization Results	Ref.
TiO_2_	solution	- Photo-oxidation by UV irradiation through an iron-halogenide lamp	- 2 M of APTES in 150 L Toluene at 80 °C for 2 h, under a dry N_2_	- Sonication in toluene for 15 min - N_2_ dry	⮚ WCA: 55 ± 5° ⮚ AFM surface roughness: 0.75 nm ⮚ XPS thickness: ∼5.3–6.5 Å	[64]
TiO_2_	solution	- Anodization (hydroxylation) in 1 M H_2_SO_4_ at 20 V for 20 min	- 0.001, 0.01, 0.1, 1.0, 10, or 100 mM of APTES in toluene (50 mL) at 25 °C, 50 °C, 70 °C or 90 °C in a water bath for 0.5, 1, 3, 5, 8 or 24 h.	- Rinsed with toluene, followed by acetone and ethanol, - Bake at 70 °C	⮚ Longer incubation times (>8 h) at lower APTES concentrations (0.01-10 mM) formed an optimal stable monolayer surface coverage. ⮚ Different attachment modes of APTES confirmed with XPS	[86]
SiO_2_	solution	- Piranha treatment 45 min - Rinse with H_2_O - Bake at 110 °C for 30 min	- 0.5 mL of APTES in 25 mL toluene inside Schlenk flask under N_2_ purge at 70 °C for 24 h	- Rinse with toluene (x2), ethanol (x2), and water (x2) - Bake at 110 °C for 15 min	⮚ Thicker and more variable silane layers with more moisture ⮚ Ellipsometry: thickness 23 Å, after hydrolysis 8 Å ⮚ WCA: 32°	[44]
SiO_2_	Solution	- Piranha treatment 30 min - Rinse with H_2_O - Dry with N_2_ gas	- 1, 10, or 33% APTES in toluene in a sealed vial at 25 °C or 75 °C for 1, 24, or 72 h	- Rinse with toluene, methanol, and water	⮚ AFM surface roughness: 0.53 nm (at 1% APTES, 1 h incubation) ⮚ Ellipsometry thickness: 1.5 nm ⮚ WCA: 60–68° ⮚ Increasing the incubation time increased the APTES film thickness ⮚ Film quality did not show strong temperature dependence for 1 h reaction	[71]
SiO_2_	Solution	- 1:1 HF/H_2_O solution - Standard SC1/SC2 RCA cleaning method	- 0.1% solution of APTES in toluene inside a dry nitrogen-purged glovebox at room temperature, 50 °C, and 70 °C for 2 min-48 h	- Rinse with toluene - Sonicate in toluene for 5 min	⮚ Higher temperatures exhibited denser packing of the propyl chains ⮚ More covalent bond formation between the APTES molecules for treatment at 70 °C 49 min ⮚ Surface thickness: 1.8 nm, 2–2.5 layers of monolayer ⮚ AFM surface roughness: 0.3 nm ⮚ Hydrolytic stability: 50% degradation of the films after 6 h	[53]
SiO_2_	Solution	- Piranha treatment 15 min - Rinse with H_2_O - Dry with N_2_ gas - Oxygen plasma 10 min - Rinse with H_2_O - Dry with N_2_ gas - Bake at 110 °C for 1 h	- 2% APTES in toluene at 100–120 °C for up to 12 h	- Rinse with toluene - Bake at 110 °C for 1 h	⮚ Monolayer of silane: 2% APTES, incubation for 1 h ⮚ WCA: 63° ⮚ AFM surface roughness: 0.69 nm	[10]
SiO_2_	Solution	- 3:7 solution of ethanol to 10 M sodium hydroxide for 30 min on an orbital shaker - Rinse with H_2_O - Dry with N_2_ gas - Piranha treatment 30 min - Rinse with H_2_O - Dry with N_2_ gas	- 1% APTES in toluene at room temperature and at 70 °C - 1/500 dilution of stock solution (50% methanol, 47.5% APTES and 2.5% nanopure water) in methanol	- Rinsed with toluene - N_2_ dry - Bake at 70 °C 30 min	⮚ Two- and three-dimensional polymer networks observed in toluene solutions at room temperature and at 70 °C. Room T incubation: loss of APTES films in the presence of both the buffer and protein Spectroscopic ellipsometry thickness: 26 Å and 26.6 Å, respectively Hydrolytic stability: 21 h/8 Å and 1 h/3.7 Å, respectively AFM surface roughness:0.3 nm and 19.99 Å, respectively ⮚ APTES monolayer was observed from methanol-based stock solution. Spectroscopic ellipsometry thickness: 8 Å AFM surface roughness: 0.2 nm Hydrolytic stability: 1 h/3.7 Å; WCA: 45–60 °	[65]
SiO_2_	Solution	- Piranha treatment 1 h - Rinse with H_2_O - Bake 110 °C 30 min	- 0.5 mL APTES in 25 mL toluene under N_2_ at 70 °C for 3 h	- Rinse with toluene (2x times), ETOH (2x times), and water (2x times) - Bake at 110 °C for 15 min	⮚ Trace amount of water present in toluene and on glassware was sufficient for the APTES layers formation. ⮚ Monolayer formation at 3 h: 10 Å thickness; WCA: 38–43° ⮚ Hydrolytic stability: complete loss of attached silane layers upon water immersion i.e., not stable ⮚ APTES layers prepared at longer silanization times were thicker due to the formation of multilayers: 19 h: 57 Å; WCA: 15–22° ⮚ Hydrolytic stability: complete loss of attached silane layers upon water immersion	[5]
SiO_2_	Solution	- Sonication in acetone 10 min, acetone/ethanol 50/50 for 10 min - Rinse with H_2_O - Piranha treatment 15 min - Rinse with H_2_O - Dry with N_2_ gas - UV-ozone cleaner 2 h	- 50 mM of APTES in toluene immersion times varying from 1 to 24 h at 90 °C and room temperature	- Sonicated for 10 min in anhydrous toluene - N_2_ dry - Bake at 90 °C for 2 h	⮚ IR spectra analysis: amine terminal groups and protonated amine groups were present after APTES treatment ⮚ WCA: 63–65° ⮚ Hydrolytic stability/AFM surface roughness: Room T incubation: loss of APTES films in the presence of both the buffer and protein solutions measured between 10 min and 12 h/3.14 nm, many agglomerates 90 °C incubation: no significant detachment/1.28 nm, no agglomerates	[45]
SiO_2_	Solution	- Plasma cleaning 2 min	- APTES (1–4%) in either 96% ethanol or 1 mM acetic acid in deionized water - Incubated for 10–60 min	- NA	⮚ AFM surface roughness: 1 mM acetic acid: 0.1 nm, did not promote extensive APTES self-polymerization, uniform coverage 96% ethanol: 0.08–0.55 nm for incubations 10–60 min, ⮚ Silanization of ring resonator chips: 1 mM acetic acid: change in resonance frequency was initially quick, followed by a less rapid increase, after final rinse the change was similar for all concentrations of APTES (1–4%); monolayer 96% ethanol: smaller initial change, followed by a more strongly time-dependent increase, after final rinse the change was proportional to concentrations of APTES (1–4%); multilayer	[49]
SiO_2_	Solution	- Piranha treatment 20min - Rinse with H_2_O - Dry with N_2_ gas	- 2–5% APTES in absolute ethanol for 20 min at room temperature	- Rinse with distilled water - N_2_ dry - Bake 120 °C for 20 min	⮚ Maximum plateau fluorescence signal values were obtained for APTES concentration in water equal to or greater than 2% (v/v), and APTES concentration in ethanol equal to or greater than 5% (v/v). ⮚ 20-min incubation was optimal for the aqueous APTES protocol and a 60-min incubation for APTES in ethanol	[9]
SiO_2_	Solution	- 2% (w/v) SDS treatment for overnight - Rinse with H_2_O - Dry with N_2_ gas - UV-ozone cleaner 10 min at 50 °C - Immersion in CH_3_OH/HCl (1:1) mixture for 30 min at room temperature	- 1–10% of APTES in ethanol for 20 min and 60 min	- Rinse with 6% acetic acid for 20 min	⮚ XPS: N was three times and C were two times larger i.e., more than 1 monolayer of APTES. ⮚ increasing the APTES concentration and reaction time did not significantly influence the multilayer formation. ⮚ 6% acetic acid effectively removed the APTES multilayer, leaving the surface with a monolayer APTES characteristic.	[7]
SiO_2_	Vapor (YES CVD)	- Washed with soap and water, 2-propanol, and acetone - Dry with N_2_ gas - Plasma cleaning/activation - N_2_ purge	- APTES introduction at 150 °C for 5 min	- 3 cycles of N_2_ gas purges	⮚ Surface thickness: 0.66 ± 0.05 nm ⮚ AFM surface roughness: 0.152 ± 0.005 nm ⮚ WCA: 44° ⮚ Stability in pH 10 buffer: 20% nitrogen loss after 2 h ⮚ 30–35% nitrogen loss after 4 h	[12]
SiO_2_	Vapor (YES CVD)	- Oxygen plasma cleaning/activation for 10 min - Surface hydration with 500 L of water	- 500 L of APTES introduced under half-atmosphere N_2_ (500 Torr) at 150 °C for 10 min	- Several N_2_ purges	⮚ Surface thickness: 0.42 ± 0.03 nm ⮚ AFM surface roughness: 0.22 nm ⮚ WCA: 40 ± 1° ⮚ Hydrolytic stability: 50–70% of APTES layer retained after 1 h	[53]
SiO_2_	Vapor (YES CVD)	- Cleaned/hydroxylated with piranha solution at 85 °C for 30 min - Dry with N_2_	- 2 mL of APTES introduced at 150 °C for 30 min	- NA	⮚ Surface thickness: 0.65 ± 0.04 nm ⮚ AFM surface roughness: 0.239 nm ⮚ WCA: 44 ± 2°	[89]
SiO_2_	Vapor (CVD)	- Piranha solution submersion for 45 min - Water rinse - Dry in an oven for 30 min at 110 °C	- Under N_2_ gas, 500 L of APTES for 24 h at 70 and 90 °C and 48 h at 90 °C	- Rinse with toluene, ethanol, and water - Dry in 110 °C oven for 15 min	⮚ 24 h, 70 °C Surface thickness: 0.6 ± 0.1 nm ⮚ 24 h, 90 °C Surface thickness: 0.5 ± 0.1 nm WCA: 50° ⮚ 48 h, 90 °C Surface thickness: 0.5 ± 0.1 nm WCA: 51° ⮚ All samples reduced to 0.3 nm thickness after 24 h in water, indicating a low-density monolayer	[14]
SiO_2_	Vapor (MLD)	- Piranha solution submersion for 30 s - DI water rinse - Dry with N_2_	- 5, 10, 25 sec APTES and water vapor pulses with N_2_ carrier gas at 50 sccm	- 60 s N_2_ purge - Hydrolysis for 12 h	⮚ Five pulses Surface thickness: 0.8 ± 0.1 nm WCA: 34 ± 1° AFM surface roughness: 0.176 nm ⮚ Ten pulses Surface thickness: 0.7 ± 0.1 nm WCA: 47 ± 2° AFM surface roughness: 0.172 nm ⮚ 20 pulses Surface thickness: 0.7 ± 0.1 nm WCA: 47 ± 1° ⮚ AFM surface roughness: 0.186 nm	[88]
SiO_2_	Vapor (MLD)	- 5 min acetone ultrasonic bath - 5 min IPA ultrasonic bath - Cleaning/activation with oxygen plasma	- 300 L of APTES introduced via argon carrier gas at 27 sccm; process pressure of 0.375 Torr and reaction time of 10 min	- Argon removed and pressure reduced to 75*10^−7^ Torr for 24 h	⮚ Surface thickness: 0.71 nm ⮚ WCA: 63.9°	[66]

**Table 3:** APTES grafting methods and characterization results on NP’s surface.

Oxide Surface	Solution vs Vapor Phase Deposition	Surface Pre-Treatment	APTES Deposition	Post-Treatment	APTES Monolayer Characterization Results	Ref.
Fe_2_O_3_ NPs	Solution	- NA	- NP’s dispersed in water (100 mL) and then APTES (0.2 or 2 mL) was added, stirring at 30 or 70 °C. - NP’s dispersed 100 mL in methanol, APTES (0.2 or 2 mL) was added, stirring at 30 or 70 °C under N_2_.	- Rinse with ethanol - Rotary evaporation dry - Bake 60 °C for 24 h	⮚ Thermal gravimetric analysis: estimation of silane grafting densities (Dg); at 70 °C: Dg increased within the first 1h incubation for 0.2 and 2% APTES; at 30 °C: Dg increase was dependent on APTES concentration ⮚ The use of different solvents does not change the kinetics of the silanization process, the degree of silanization depended greatly on the reaction conditions	[80]
ZnO NPs	Solution	- ZnO NPs were dispersed into distilled water (50 mL, pH 6.5), stirred for 1 h	- 1 mL of APTES was added (under Ar flow for toluene) - pH was increased to 9.7 (or 10.8) and after a few minutes was stabilized to 8.9–9.2 (or 10.4–10.6) for the acid/base condition, stirred for 24 h (under Ar flow for 15 h for toluene)	- Filtration, rinse with alcohol and acetone. - Bake at 60 °C under a vacuum	⮚ XRD and SEM: APTES coating plays a role in growth inhibitor of NPs ⮚ Acid process: fast grafting controlled by the condensation process. ⮚ Basic process, the starting concentration of silane is an important parameter in controlling the silica contents and the grafting. ⮚ Toluene process, the grafting is controlled by the amount of water on the ZnO nanoparticle surface. ⮚ Photostable NPs	[92]
		- Dispersed into anhydrous toluene (200 mL) under argon flow, stirred for 1 h		- Filtration, rinse with toluene - Bake at 110 °C for 2 h		
Fe_2_O_3_ NPs	Solution	- Rinse with methanol and water in the presence of a permanent magnet - Dry in a vacuum desiccator	- NP’s dispersed 100 mL in methanol, 2% APTES, shaking 24 h at 70 °C	- Rinse with methanol and water in the presence of a permanent magnet - Dry in a vacuum desiccator	⮚ FTIR analysis: coverage with silane, presence of amine groups, conjugation to a drug ⮚ Ninhydrin colorimetric assay: fractional monolayer coverage 96.6% ⮚ XPS analysis: interlinking silane molecules on the NP surface	[83]

## Data Availability

Not applicable.
